# Decreased Brain Neurokinin-1 Receptor Availability in Chronic Tennis Elbow

**DOI:** 10.1371/journal.pone.0161563

**Published:** 2016-09-22

**Authors:** Clas Linnman, Ciprian Catana, Kurt Svärdsudd, Lieuwe Appel, Henry Engler, Bengt Långström, Jens Sörensen, Tomas Furmark, Mats Fredrikson, David Borsook, Magnus Peterson

**Affiliations:** 1 Center for Pain and the Brain, Department of Anesthesiology, Perioperative and Pain Medicine, Boston Children’s Hospital, Harvard Medical School, Boston, MA, United States of America; 2 Martinos Center for Biomedical Imaging, Massachusetts General Hospital, Harvard Medical School, Boston, MA, United States of America; 3 Department of Public Health and Caring Sciences, Family Medicine and Clinical Epidemiology, Uppsala University, Uppsala, Sweden; 4 Uppsala PET Centre, Department of Radiology, Oncology and Radiation Sciences, Uppsala University, Uppsala, Sweden; 5 Uruguayan Centre of Molecular Imaging (CUDIM), Faculty of Medicine and Faculty of Sciences, University of the Republic, Montevideo, Uruguay; 6 Department of Biochemistry and Organic Chemistry, Uppsala University, Uppsala, Sweden; 7 Neuropsychopharmacology Section, Faculty of Medicine, Imperial College, London, United Kingdom; 8 Department of Psychology, Uppsala University, Uppsala, Sweden; 9 Department of Clinical Neuroscience, Karolinska Institute, Stockholm, Sweden; Charite Universitatsmedizin Berlin, GERMANY

## Abstract

Substance P is released in painful and inflammatory conditions, affecting both peripheral processes and the central nervous system neurokinin 1 (NK1) receptor. There is a paucity of data on human brain alterations in NK1 expression, how this system may be affected by treatment, and interactions between central and peripheral tissue alterations. Ten subjects with chronic tennis elbow (lateral epicondylosis) were selected out of a larger (n = 120) randomized controlled trial evaluating graded exercise as a treatment for chronic tennis elbow (lateral epicondylosis). These ten subjects were examined by positron emission tomography (PET) with the NK1-specific radioligand 11C-GR205171 before, and eight patients were followed up after treatment with graded exercise. Brain binding in the ten patients before treatment, reflecting NK1-receptor availability (NK1-RA), was compared to that of 18 healthy subjects and, longitudinally, to the eight of the original ten patients that agreed to a second PET examination after treatment. Before treatment, patients had significantly lower NK1-RA in the insula, vmPFC, postcentral gyrus, anterior cingulate, caudate, putamen, amygdala and the midbrain but not the thalamus and cerebellum, with the largest difference in the insula contralateral to the injured elbow. No significant correlations between brain NK1-RA and pain, functional severity, or peripheral NK1-RA in the affected limb were observed. In the eight patients examined after treatment, pain ratings decreased in everyone, but there were no significant changes in NK1-RA. These findings indicate a role for the substance P (SP) / NK1 receptor system in musculoskeletal pain and tissue healing. As neither clinical parameters nor successful treatment response was reflected in brain NK1-RA after treatment, this may reflect the diverse function of the SP/NK1 system in CNS and peripheral tissue, or a change too small or slow to capture over the three-month treatment.

## Introduction

Pain from the tendons that join the forearm muscles on the outside of the elbow, i.e. tennis elbow (TE) or lateral epicondylitis, has a prevalence of 1–3% in the population [1,2,3]. Generally thought of as a persistent inflammatory process caused by initial overuse and/or repetitive microtrauma [4,5], the cause of pain is mostly unknown [6,7]. The unaffected arm also displays cold, heat and pressure hypersensitivity, indicating that both peripheral and central mechanisms may be involved [8,9]. As such, the disease represents an interesting, unilateral and treatable condition that may lend itself to insight in the pathophysiology of more severe chronic pain. In chronic TE (defined as lasting more than 3 months), there are few inflammatory cells present, and tissue degeneration appears [7,10,11,12]. An increase of neural fibers and transmitters, including SP, has been observed in chronic TE tissue samples [13,14,15,16]. SP contributes to local neurogenic inflammation [17,18,19], promotes tissue healing [20], [21]) by enhancing inflammatory response [22]), and serves as a neuropeptide in the nociceptive pathway via its primary receptor, the neurokinin 1 (NK1) receptor [23]. NK1 receptors are widely distributed in the brain except the cerebellum [24] [25]) and are abundant in the basal ganglia, nigrostriatal pathways [24] and brain regions that are involved in stress, fear and affective response (e.g. limbic system (amygdala, hippocampus), hypothalamus and frontal cortex). [24] [26]. SP and increased expression of NK1 receptors has been demonstrated in human Achilles tendinosis [14], and we recently found that the radiolabeled NK1 receptor antagonist ^11^C-GR205171 has elevated retention in the affected regions in TE [27]. Here, we investigated central NK1 receptor availability (NK1-RA) in patients with unilateral chronic tennis elbow before and after therapeutic exercise [28]. We further sought to characterize interactions between central NK1-RA alterations, pain ratings, functional severity, symptom duration and peripheral ^11^C-GR205171 uptake in the affected limb [27].

## Materials and Methods

### Study population

The study sample was part of a larger randomized controlled trial, evaluating graded exercise as a treatment for chronic TE, see [28] for details. Further, the study sample was identical to that reported in [27], where we report peripheral uptake of ^11^C-GR205171. Briefly, the inclusion criteria included a verified TE diagnosis, symptoms for more than three months, and age 20–75 years. Subjects with concomitant supinator syndrome, compartment syndrome of the anconeus muscle, rhizopathy, inflammatory joint disease, fibromyalgia, previous elbow surgery, treatment by injection of steroids, within the previous three months, and an inability to understand Swedish were excluded. Further exclusion criteria for the PET study included the following: current medication interfering with the nervous or inflammatory system, substance abuse, pregnancy, recent or planned participation in another PET study, X-ray or other significant exposure to radiation, bilateral symptoms or other pain diagnosis of the upper extremities.

The diagnosis was verified by pain on palpation, stretching (Mill´s test), loading and Maudsley´s middle finger test by a general practitioner and pain specialist (MP). 120 subjects were included in the larger randomized controlled trial (RCT), and each subject recruited in the RCT was invited to participate in the PET study, until ten accepted. The RCT is registered as NCT00888225 at http://clinicaltrials.gov/.

For a detailed description of study participants, we refer to [27]. Briefly, five men and five women with a mean age of 48.7 years (±8.5 years) with an average duration of 52.0 (±42.9) weeks of TE pain participated in the PET study. Eight of these ten patients also participated in a second PET investigation after treatment. See Table 1 for details.

**Table 1:** Characteristics of the Patient Population.

Metric		n or mean	(SD) or %
Number of participants		10	
Age, years		48.7	(8.5)
Women		5	50%
Educational level			
	Compulsory education only	2	20%
	Vocational training	4	40%
	Upper secondary school	1	10%
	College or university	3	3%
Marital status			
	Never married	1	10%
	Married or cohabiting	9	90%
Smoking habits			
	Never smoked	5	50%
	Ex-smokers	3	30%
	Current smokers	2	20%
Duration of present epicondylosis, weeks		52.0	(42.9)
Previous treatments given			
	NSAID	4	40%
	Acupuncture	4	40%
	Steroid injections	3	30%
	Stretching	4	40%
	Orthosis or other fixative	3	30%
	Massage	1	10%
	Rest	1	10%
	No previous treatment	1	10%
VAS 0–100 Pain ratings			
	Unaffected elbow pre-treatment, n = 10	5	(2)
	Affected elbow pre-treatment, n = 10	58	(10)*
	Unaffected elbow post-treatment, n = 8	4	(2)
	Affected elbow post-treatment, n = 8	25	(21)**

n = number of participants, SD = Standard Deviation, NSAID = Nonsteroidal anti-inflammatory drug, VAS = Visual Analog Scale.

* Significantly higher pain ratings in affected elbow, paired t-test p<0.001.

** Significant reduction of pain after treatment, paired t-test, p = 0.001.

In addition, eighteen healthy, pain-free control subjects (9 females, 9 males, age 35 ± 9 years) were recruited through advertisements. Data from this control group have previously been reported in [29] and [30]. All subjects gave written informed consent before entering the study. The Regional Ethical Review Board in Uppsala, Sweden and the Radiation Safety Committee in Uppsala, Sweden approved the study

### Treatment procedure and clinical parameters

All subjects, except one, had right lateralized TE. Treatment consisted of a three-month daily exercise regime performed at home, with gradually increasing load on the extensor muscles of the affected forearm, see [28] for details. For both PET examinations (see below), patients rated their pain on a 100 mm visual analogue scale (VAS) during maximum voluntary contraction of the forearm extensor muscles. General arm function was measured at baseline and after 3 months of treatment using the Disability of Arm, Shoulder, and Hand questionnaire (DASH) [31,32]. DASH contains 30 questions on the ability to perform activities, using a five-degree Likert scale ranging from ‘no problem’ to ‘impossible’.

### PET examination

The PET examinations were performed twice in eight of the ten patients, before and after the treatment protocol of the RCT. Two patients declined a second PET examination after treatment and thus only participated once in the PET examination. PET examinations were performed only once in the healthy controls.

**Fig 1:**
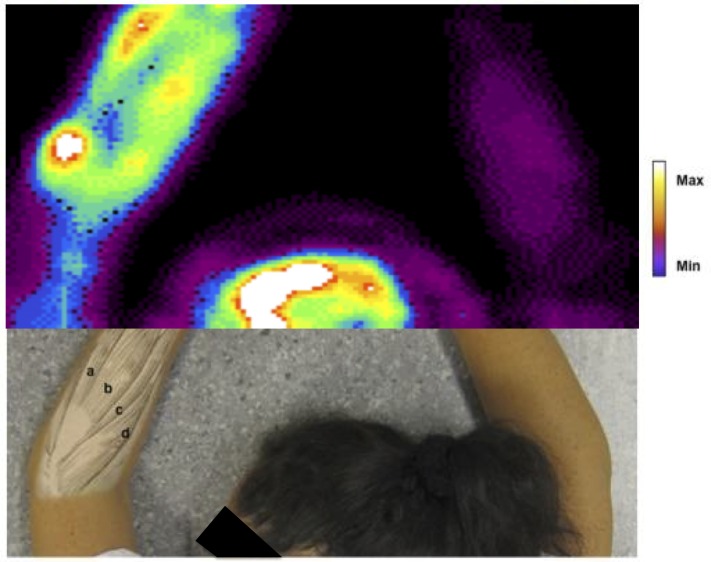
Elevated Peripheral Uptake of ^11^C-GR205171 in a Patient with Left-Sided Tennis-Elbow Pain. Fig adapted from [27]. The subject has given written informed consent, as outlined in the PLoS consent form, for the publication of the photograph.

The NK1-specific radioligand ^11^C-GR205171, synthesized according to standard manufacturing procedures [33] at the Uppsala PET center was injected as a bolus, and PET scanning was performed on a Siemens ECAT EXACT HR+ whole body tomograph (CTI, Knoxville, TN, USA). The scanner enables acquisition of 63 contiguous planes of data with 2.46 mm plane spacing resulting in a total axial field of view of 155 mm. Subjects were instructed to rest quietly and relax during the investigation. Healthy controls were placed supine in the scanner, while patients were placed in prone position with their arms stretched above their heads and gently fixed, so that both the brain and, after table movement, the elbow joints of both arms could be investigated, Fig 1 and [27].

For brain imaging, a 10 min transmission scan was performed using three retractable 68Ge rotating line sources. Thereafter, a bolus of approximately 5.2 MBq/kg bodyweight (average dose 400 ± 13 MBq), diluted in saline, total volume 5 ml was injected into the dorsal vein of the right foot, and 14 frames of dynamic data with progressively increasing duration (4 × 60 s, 3 × 120 s, 7 × 300 s), in total 45 min, were collected in 3D mode starting at the time of bolus injection. After the brain scan, the patients were moved and a 10-minute frame was collected over the elbow joints [27]. In the control group, additional frames were collected over the brain. These were discarded in the current analysis to allow for comparison between patients and controls. The PET data were reconstructed to a 128 × 128 matrix with filter back projection, corrected for photon attenuation, decay, scattered radiation, and random coincidences according to standard procedures [34].

### PET tracer modeling

Dynamic ^11^C-GR205171 PET frames were realigned within scans to adjust for movements during scanning. Parametric PET images were generated using the Patlak reference tissue model [35] with the cerebellar cortex as a reference region for the time window between 20 and 45 minutes post-injection. The resulting Patlak image reflects neurokinin-1 receptor availability (NK1-RA). To further examine tracer uptake, we calculated Standardized Uptake Values (SUV) (i.e. adjusted for injected dose and weight) for all subjects and extracted time radioactivity curves.

### Data analysis

All except one subject had right-sided TE. It can be assumed that unilateral chronic pain mostly affects the contralateral hemisphere; thus, the brain data of the one patient with left TE was flipped in the left-right direction to homogenize the data with regards to laterality, as has been done in other clinical pain studies [36,37,38,39,40]. Based on the animal literature on NK1 receptor alterations in chronic pain, and on our previous findings in chronic whiplash disorder [29], region of interest (ROI) analyses were performed on the bilateral insula, ventromedial prefrontal cortex (vmPFC), the postcentral gyrus, the anterior cingulate, thalamus, caudate, putamen and amygdala. A midbrain (including periaqueductal gray) ROI was also defined. For control regions, ROI´s were defined for the whole brain, the left and right cerebellum, and the visual cortex (Brodmann area 17,18 & 19). All ROI´s were defined from the AAL library [41], except for the midbrain (a 5 mm radius sphere at MNI_xyz_ (0, -10, -30) [42]), the vmPFC (two 10 mm radius spheres at MNI_xyz_ (±8, 36, -18) [29]), and the cerebellum (two 10 mm radius spheres at MNI_xyz_ (±28, 72, -45).

The average NK1-RA values from all voxels included in the ROIs were extracted and t-tests were performed between healthy subjects and controls, between left and right hemispheres within the patient group, and finally within patients pre- and post-treatment. With a total of 21 ROI´s, the Bonferroni corrected P value of 0.05 was set at p<0.0024.

In addition to ROI analyses, NK1-RA differences between patients and controls were analyzed on a voxel by voxel basis using statistical parametrical mapping (SPM8) in a general linear model (GLM) with age and gender as nuisance variables. In four separate SPM8 GLM regression models, we further explored potential correlations between central NK1 receptor alterations and *i)* pain, *ii)* functional severity, *iii)* symptom duration and *iv)* peripheral ^11^C-GR205171 uptake in the affected limb [27]. For all SPM analyses, the cluster significance level was set at p<0.05 family wise error corrected.

### Treatment responses

NK1-RA alterations after treatment were evaluated in the 8 available patients in a ROI analysis with paired t-test, and in a whole brain GLM with paired t-tests. Moreover, treatment-induced changes in pain ratings and in peripheral uptake of ^11^C-GR205171 were evaluated in relation to treatment-induced changes in CNS uptake of ^11^C-GR205171.

## Results

### Pain ratings, disability and treatment response

Pain was rated on a 100 mm visual analogue scale (VAS), during maximum voluntary contraction of the forearm extensor muscles. Patients rated their pain in the affected arm an average 58 (±10) and in the unaffected arm an average of 5 (±2), before treatment (p<0.0001). After treatment, patients rated their pain in the affected arm an average of 25 (treatment effect p = 0.007). Treatment also led to a significant reduction in disability (DASH change = 11 points, p = 0.001).

Seven of the eight subjects had substantial (39–96%) reductions in VAS ratings after treatment, while one subject essentially rated pain the same before and after treatment (VAS_pre_ = 68, VAS_post_ = 67).

### Central neurokinin-1 receptor availability

**Fig 2:**
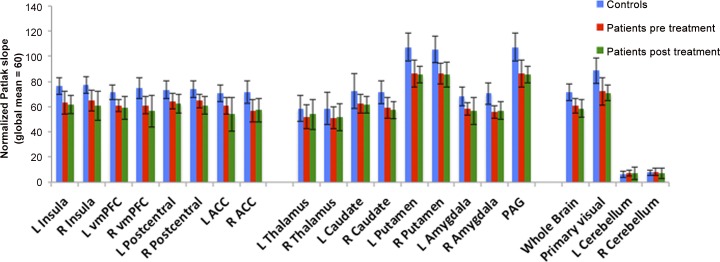
Average Patlak Values Signifying NK1-RA for Regions of Interest Across All Healthy Controls. (n = 18, in blue), patients pre-treatment (n = 10, in red) and patients post-treatment (n = 8, in green).

Before treatment, patients had significantly lower NK1-RA in most ROIs (insula, vmPFC, right postcentral gyrus, anterior cingulate, right caudate, putamen, amygdala and the midbrain). There were no group differences in the thalamus. With regards to the control regions, there were no group differences in the cerebellum (Patlak reference region with no NK1 receptors), but both whole brain and primary visual cortex NK1-RA were significantly lower in the patient group, see Table 2 and Fig 2.

**Table 2:** Region of Interest Analysis. Average NK1-RA (Normalized Patlak Slope Values) in Regions of Interest. HC vs. Pat Indicates t-test Between 18 Healthy Controls and 10 Patients with Chronic Tennis Elbow. Pat_pre_ vs. Pat_post_ Indicates Paired t-tests in 8 Patients Examined Before (Pre) and After (Post) a Three-months Treatment Program of Daily Exercise.

ROI	Healthy (stdev)	Patients Pre	Patients post	HC vs. Pat	Pat_pre_ vs. Pat_post_
*Cortical regions*					
L Insula	76.5(6.8)	63.3(9.2)	61.9(7.4)	0.00020*	0.14
R Insula	77.4 (6.7)	64.9(8.3)	60.8(11.7)	0.00019*	0.12
L vmPFC	71.4(5.7)	61.0(4.8)	59.2(9.3)	0.000042*	0.54
R vmPFC	74.6(8.4)	61.1(6.7)	56.5(12.7)	0.00018*	0.13
L Postcentral	73.5(7.2)	64.5(6.3)	62.1(7.4)	0.0028	0.045
R Postcentral	74.0(6.9)	64.5(5.7)	61.2(7.1)	0.0011*	0.057
L ACC	70.4(6.7)	60.8(6.8)	53.9(13.3)	0.0014*	0.18
R ACC	71.5(8.8)	57.0(9.1)	57.3(9.4)	0.00035*	0.24
*Subcortical regions*					
L Thalamus	58.4(10.3)	51.8(9.5)	53.8(11.9)	0.11	0.93
R Thalamus	58.5(12.8)	51.2(8.7)	51.5(10.7)	0.12	0.58
L Caudate	72.6(14.0)	62.5(7.7)	61.3(6.6)	0.045	0.39
R Caudate	71.5(9.4)	59.0(8.3)	57.4(7.0)	0.0017*	0.070
L Putamen	107.4(11.0)	86.2(11.0)	85.4(6.4)	0.000046*	0.42
R Putamen	105.7(10.7)	86.4(8.3)	85.4(9.9)	0.000042*	0.45
L Amygdala	68.0(7.3)	58.3(5.2)	56.5(10.8)	0.0011*	0.52
R Amygdala	70.6(8.7)	55.9(5.0)	56.7(7.0)	0.000049*	0.94
Midbrain	107.4(11.0)	86.2(11.0)	85.4(6.4)	0.000046*	0.42
*Control regions*					
Whole Brain	71.2(6.6)	60.5(5.7)	58.4(7.1)	0.00021*	0.090
Primary visual	88.4(10.0)	72.2(11.0)	70.9(6.3)	0.00050*	0.26
L Cerebellum	6.2(2.3)	7.2(2.3)	7.1(4.8)	0.26	0.92
R Cerebellum	7.6(2.4)	8.4(3.1)	7.1(4.4)	0.45	0.47

L left, R Right, vmPFC ventromedial Prefronal Cortex, ACC Anterior Cingulate Cortex

* Significant at p<0.0024 (Bonferroini corrected p<0.05)

**Fig 3:**
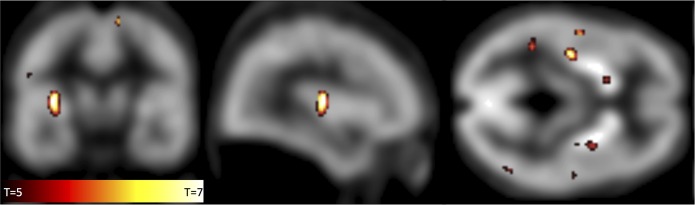
Regions with Significantly Lower NK1-RA in the Patient Group Pre-Treatment, as Compared to the Healthy Group, at a Family Wise Error Corrected p-value of <0.05 in the Insula. The color bar indicates t-values. The background image is the average Patlak-slope value of all patients and controls (note the absence of NK1-receptors in the cerebellum).

The voxelwise SPM analysis indicated that the most pronounced NK1-RA alterations (i.e. lower NK1-RA in patients) occurred in the posterior insula, contralateral to the injured elbow, see Table 3 and Fig 3. There were no significant correlations between brain NK1-RA and *i*) pain ratings, *ii*) functional severity, *iii*) symptom duration or *iv*) peripheral ^11^C-GR205171 uptake (data from [27]) in the affected limb.

**Table 3:** SPM Analysis Results. Significant Reductions in NK1-RA in the Patient Group as Contrasted to the Healthy Control Group when Controlled for Age and Gender.

Region	Cluster size	Peak P_FWE_	T-value	MNI_X,Y,Z_
Left Insula	61	0.019	5.2	-36, -18, 5
Right ParaHippocampal Gyrus	141	0.001	4.91	30, 2, -37
(Right Middle Temporal Pole)			4.6	(42, 4, -27)
(Right Fusiform Gyrus)			3.71	(36, -6, -37)
Left Superior Temporal Pole	160	0.001	4.86	-28, 8, -25
Left Superior Occipital Gyrus	44	0.041	4.54	-24, -78, 37
Left Superior Temporal Gyrus	67	0.015	4.5	-50, -44, 13
Left Hippocampus	51	0.029	4.43	-12–4–13
(Left Putamen)			3.95	(-20, 6, -5)
Right Superior Temporal Gyrus	63	0.017	4.31	70, -44, 23
(Right Supramarginal Gurus)			3.92	(70, -48, 33)

Cluster subpeaks are indicated in parenthesis, labelling based on AAL-library. FWE family wise error corrected p value.

### Time radioactivity curves

**Fig 4:**
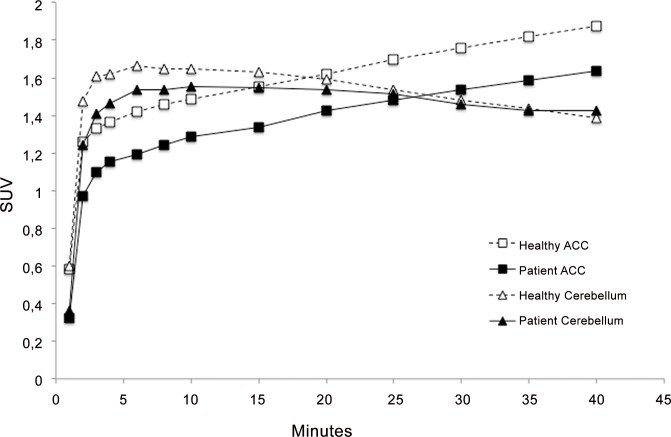
Average Time Radioactivity Curves for the Anterior Cingulate (ACC) and the Cerebellum for Healthy Subjects (n = 18, solid lines) and Patients Pre-Treatment (n = 10, dashed lines). X-axis is in seconds; y- axis is Standardized Uptake Values (normalized for dose and weight).

^11^C-GR205171 time radioactivity curves were similar in the cerebellum region, and decreased in patients across cortical and subcortical regions, see Fig 4 for an illustration of time radioactivity curves in the anterior cingulate and cerebellum.

### Treatment effects

In seven out of the eight subjects examined after treatment, pain ratings decreased substantially (39 to 96%), but there were no significant changes in NK1-RA after treatment, either in the ROI analysis or in the SPM analysis. Furthermore, there were no significant correlations between changes in CNS NK1-RA and changes in pain ratings or peripheral tracer uptake.

## Discussion

We observed a general reduction of NK1-RA throughout the CNS in patients with chronic tennis elbow. This is in line with previous human studies on whiplash associated disorder [29] and an animal model that showed decreased hippocampal NK1 receptor gene expression in response to peripheral inflammation [43,44]. Moreover, NK1-RA levels were most reduced in the posterior insula cortex contralateral to the TE-arm, suggesting that the NK1-RA levels may be affected in a somatotopically representative manner. Ascending sensory pathways conveying proprioceptive and somatosensory information about the body’s internal state terminate in the posterior insula [45]. Accordingly, the insula plays a key role in pain processing: For example, the posterior insula functional activation to muscle pain (without cutaneous sensation) [46], and treatment reversible gray matter volume reductions of the insula have been reported in hip osteoarthritis patients [47]. In a study on irritable bowel syndrome [48] treatment with a neurokin-1 receptor antagonist reduced pain induced functional activation of the posterior insula (and other regions). However, further studies are needed before the observed alterations can be linked to pain-specific roles (see [49] for a discussion on pain-specificity in the posterior insula). The present findings are difficult to directly attribute to the clinical condition, as NK1-RA levels were also reduced in regions not directly involved in pain processing, like the visual cortex, and did not correlate with pain ratings, symptom duration, peripheral inflammation or treatment response. Cortical NK1-tracer binding capacity has in previous investigations been noted high in the primary visual cortex [24]. Our findings may reflect the diverse function in the SP-NK1 system, and a general decrease in brain NK1-RA. This lack of relation to clinical symptoms may also be due to the relatively small sample size or it may reflect a true absence of clinical validity.

In an animal model with voluntary, highly repetitive, negligible force reaching task—similar to that of a tennis elbow repetition injury—SP levels and spinal cord NK1 receptor expression was up-regulated in the superficial lamina of spinal cord dorsal horns at 6 and 10 weeks. [50]. Other animal models have in a similar manner shown increased NK1 receptor gene expression in the dorsal horn in response to peripheral inflammation [43,51], while hippocampal NK1 receptor gene expression was simultaneously decreased [43]. This suggests the SP-NK1 receptor system modulates inflammation, nociception and CNS perception in a differential manner in peripheral tissue, spinal cord and brain [43,52]. It has been a matter of discussion whether ^11^C-GR205171 can be displaced by endogenous SP [6; 39]. In the present study, decreased availability of NK1 receptors could imply an increased synaptic level of endogenous SP, a decreased number of NK1 receptors or a combination.

Of note, the included patients all displayed peripherally elevated uptake of ^11^C-GR205171 in the affected arm [27], but there was no correlation between peripheral uptake and central NK1-RA. This may, at least partly, be explained by differential modulation in peripheral tissue and brain [43]. Despite promising results in rodents, systemic blockade of NK1 receptors in human beings has not shown any convincing analgesic effect [53]. Transient NK1 receptor availability has been suggested as one possible explanation, as the NK1 receptor can be desensitized [54]as well as internalized [54,55,56]. As the activation of NK1 receptors has different effects in peripheral tissue [20,21], spinal cord [23,51]and brain [24,26], it is possible that systemic blockade of the NK1 receptor may have only indirect effects on nociception and perception of pain. Overlapping pathways for signal transduction may be another explanation of why blockade of only the NK1 path does not have a significant clinical effect [53]. In humans there are several overlapping pathways for pain signaling, which may reflect the phylogenetic evolution of a robust sensory system.

## Conclusions

The findings of this study suggest a role for the NK1 receptor in the CNS in a chronic, soft tissue-related pain condition such as chronic TE. The observed lower NK1 receptor availability in patients is interpreted as reflecting elevated endogenous SP levels creating receptor occupancy, desensitization and internalization, NK1 receptor downregulation, or a combination between these mechanisms. The CNS alterations were not specific to pain processing regions, and no effects of a three-month successful treatment program were observed, suggesting that the alterations are neither specific nor directly reflect the perception of pain.
